# Vaccination Rates in Patients with Chronic Inflammatory Skin Diseases and Immunomodulatory Systemic Therapies—Vaccinations against SARS-CoV-2, Influenza Virus or Varicella Zoster Virus

**DOI:** 10.3390/life14091157

**Published:** 2024-09-12

**Authors:** Brigitte Stephan, Anna Meineke, Matthias Augustin, Christina Sorbe

**Affiliations:** Institute for Health Services Research in Dermatology and Nursing (IVDP), University Medical Center Hamburg-Eppendorf (UKE), 20251 Hamburg, Germany; a.meineke@uke.de (A.M.); m.augustin@uke.de (M.A.); c.sorbe@uke.de (C.S.)

**Keywords:** psoriasis, COVID, zoster, flu, vaccination, prevention, immunocompromised

## Abstract

Introduction: The national guidelines and the Standing Committee on Vaccination (STIKO) of the Robert Koch Institute (RKI) in Germany support preventive vaccinations for patients under immunomodulatory treatments. Material and methods: Retrospective analysis of data from patients with chronic inflammatory skin diseases from December 2021 to December 2022 with a focus on preventive vaccinations against influenza virus, varicella zoster virus, or SARS-CoV-2. Results: Patients with chronic inflammatory skin diseases were referred to our university outpatient’s clinic for recommendations of systemic therapy. Vaccinations against influenza virus, varicella zoster virus, or SARS-CoV-2 were documented in 7365 analyzed patient files. A total of 79.7% were completely vaccinated against SARS-CoV-2, 49.7% patients were vaccinated against the influenza virus, and only 9.2% were completely vaccinated against varicella zoster virus. Discussion: In our patients who came for counselling before or during systemic treatment, vaccination rates against SARS-CoV-2, varicella zoster virus, or influenza virus were low. Patients age 60 and above had higher rates than the average German population of the same age, but still no satisfying protection. Conclusions: We suggest informing patients about preventive vaccination before and during systemic immunomodulatory treatments and emphasize the need for active communication in this vulnerable patient group.

## 1. Introduction

Vaccination is generally an effective measure to reduce the risk of disease. Patients receiving immunomodulatory treatment are immunosuppressed to varying degrees and benefit from preventive vaccination [1,2,3]. National guidelines and the Standing Committee on Vaccination (STIKO) of the Robert Koch Institute (RKI) in Germany support preventive vaccinations for this target group, especially vaccinations against respiratory infections, and early vaccination against the varicella zoster virus [4,5,6]. With recent pandemics, the susceptibility to respiratory infections of patients with inflammatory skin diseases under systemic immunomodulatory treatments became significantly important. With the rapid spreading of COVID-19 cases all over the world in spring 2020, medical societies tried to analyze the risk of respiratory infections in special patient groups and communicated cautious recommendations based on acute observations and experiences regarding continuing systemic medications during acute infection as well as updates of recommendations for preventive vaccinations in vulnerable patient groups [3,5,7,8,9,10]. In Germany, these patients with chronic inflammatory skin diseases form a relevant group, with about 2–3% of adults with psoriasis and 3–4% of adults with atopic dermatitis in the average population [11]. Physicians who care for patients with chronic inflammatory skin diseases show increased attention to vaccination in the preparation and management of immunomodulatory systemic therapies.

### Research Hypothesis

Patients with chronic inflammatory skin diseases under immunomodulatory treatments should have a better vaccination rate than the average population in Germany.

## 2. Materials and Methods

We conducted a retrospective analysis of visit data of patients in routine care at an outpatient clinic of the University Medical Center Hamburg-Eppendorf (UKE), Germany, from December 2021 to December 2022. Patients of these specialized visits for chronic inflammatory skin diseases had diagnoses like psoriasis, atopic dermatitis, hidradenitis, and others. With our data analyses, we focused on vaccinations against SARS-CoV-2, influenza virus, or varicella zoster virus. We compared vaccination rates of this patient cohort regarding subgroups by age, treatment, and disease severity, as well as to vaccination rates of the German population, as published by the STIKO and the RKI for the period from 2020 to 2022.

We did not reconfirm documentation of anamnestic information by requests for vaccination certificates. Although several other vaccinations were documented, we focused on the most relevant ones recommended for patients under immunomodulatory conditions. Patients starting or already on systemic therapies were considered one group for analysis for having the same recommendation for vaccination.

Descriptive statistics were performed using standard parameters: absolute and relative frequencies for categorical data, and minimum, mean, median, maximum, and standard deviation for continuous data. There was no imputation of missing values nor predefined test of a specific study hypothesis. The statistical tests performed aimed at exploration of potential differences in general.

Subgroup analyses were performed for patient baseline characteristics: by sex (male vs. female), by age (18–59 vs. 60+ years), by treatment (no systemic treatment vs. systemic treatment, biologic vs. non-biologic vs. Janus kinase inhibitor (JAK inhibitors) treatment), by diagnosis (psoriasis, atopic dermatitis, other diagnoses), and by severity of psoriasis based on the Psoriasis Area and Severity Index ((PASI) ≥ 10 vs. <10) and of atopic dermatitis based on the Eczema Area and Severity Index ((EASI) ≥ 7 vs. <7). Patients diagnosed with both psoriasis and atopic dermatitis were not compared by subgroups due to small and markedly different group size compared to other diagnosis groups. Patients with other dermatological diagnoses than psoriasis and/or atopic dermatitis were categorized into “other diagnoses” to focus on our main group of patients coming to us for assessment of possible systemic therapy. We excluded patients under the age of 18 as well as cases without documentation for assessment of vaccinations. We did not receive data for the type of vaccine respective to brand name.

Although parameters were not normally distributed, we used ANOVA for subgroup comparison at baseline because the group size was sufficient. Since variances were heterogeneous, post hoc comparison was performed using Tamhane-T2. For comparison of vaccination rates, we used Fisher’s exact test with “complete vaccination yes/no” as the dependent variable. The statistical analysis was conducted using SPSS for Windows version 27 (IBM, Armonk, New York, NY, USA).

## 3. Results

### 3.1. Patients

**Figure 1:**
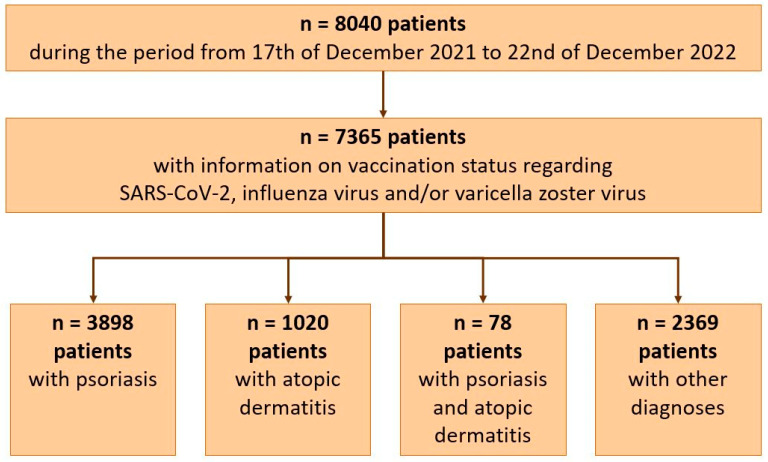
Patients included in the analysis.

Out of 8040 patients, 7365 adult patients with information on vaccination status regarding SARS-CoV-2, influenza virus, and/or varicella zoster virus could be included in the analyses: 3898 patients with psoriasis, 1020 with atopic dermatitis, 78 with both diagnoses, and 2369 patients diagnosed with other dermatological diseases, like hidradenitis suppurativa, lupus, chronic autoinflammatory wounds, and various others (Figure 1). A total of 57.6/42.3% of patients were male/female (n = 4242/3119, n = 2 non-binary, n = 2 missing data), and 75.1% were aged 18–59 (n = 5528).

Patients with psoriasis were predominantly male (60.3% vs. 39.6% female), which was a significantly higher rate than in the patients diagnosed with atopic dermatitis or other skin diseases (*p* ≤ 0.027) (Table 1). They showed a mean age of 49 years. This was similar to patients with both psoriasis and atopic dermatitis and to patients with other diagnoses than psoriasis and/or atopic dermatitis, whereas patients with atopic dermatitis were on average younger (*p* ≤ 0.010) (Table 1).

In all four subgroups, the rates for patients starting or being on systemic therapies were high, with 82.9% of the patients having psoriasis, 68.1% in the group of patients having atopic dermatitis, 69.2% of patients having both psoriasis and atopic dermatitis, and 66.1% of patients having other diagnoses. Patients with a psoriasis diagnosis were significantly more likely to receive systemic therapy (*p* ≤ 0.001).

Regarding the severity of disease at consultation, the majority of patients with psoriasis had a mild stage of the disease, similar to patients with atopic dermatitis (Table 1) and to patients with both atopic dermatitis and psoriasis. Patients with other diagnoses than psoriasis or atopic dermatitis were only scored for life quality, and 79.9% of these patients had a DLQI < 10, corresponding with a mild to moderately reduced life quality as well. The proportion of patients with a high reduction in quality of life (DLQI > 10) was highest in atopic dermatitis patients (Table 1).

### 3.2. Vaccination in All Patients of Our Study Population

Regarding the entire study population with information about vaccination against SARS-CoV-2, influenza virus, or varicella zoster virus, 49.7% of all patients had at least one seasonal vaccination (one dose 2021 or 2022) against influenza virus (n = 2862), 79.7% (n = 5869) of patients had a documented completed basic vaccination against SARS-CoV-2 (two doses) with at least one additional booster, and only 9.2% (n = 436) of patients had been completely vaccinated against varicella zoster virus (two doses). There were higher rates of vaccinations in the older study population (adults 60 years and older), with 73.8% (n = 1041) of patients with at least one seasonal vaccination against influenza virus, 89.9% (n = 1652) being fully vaccinated against SARS-CoV-2, and 22.3% (n = 254) having completed the vaccination against varicella zoster virus with two doses (Table 2).

### 3.3. Vaccination in Patients with Psoriasis

Focusing on the subgroup of patients with psoriasis and information about their vaccination status, 87.4% of these patients were completely vaccinated against SARS-CoV-2, with two basic vaccinations and at least one additional booster. A total of 9.5% of the patients with psoriasis had at least one shot. Only 3.1% of the patients had no vaccination against SARS-CoV-2. These rates were significantly higher with older patients age 60 and above compared to younger patients (*p* ≤ 0.001), but lower for patients with higher clinical severity of skin inflammation (PASI ≥ 10) (*p* ≤ 0.007). Regarding systemic medications, 100.0% of patients with JAK inhibitors were completely vaccinated against SARS-CoV-2, although this group was small in numbers (18 of 3232 patients with systemic therapy). For patients with systemic biological medication, the rate of completed SARS-CoV-2 vaccination was 87.8%, and systemic therapies other than biologics or JAK inhibitors had an even higher rate. Summarizing all vs. no systemic treatment, patients showed higher vaccination rates against SARS-CoV-2 under systemic treatment (*p* = 0.014).

In the same subgroup of patients with psoriasis and information about their vaccination status, 49.3% of the patients had at least one preseasonal vaccination against influenza virus, with 50.7% without vaccination against seasonal influenza virus. Patients aged 60 and above had a significantly higher vaccination rate (*p* = 0.001). Vaccination rates against influenza virus did not differ significantly between subgroups regarding disease severity (*p* = 0.058) and systemic treatment (*p* = 0.962). Regarding the type of systemic medication, patients with JAK inhibitors had higher rates than patients under biologics or systemic therapies other than biologics or JAK inhibitors for at least one preseasonal influenza virus vaccination.

The vaccination rates for varicella zoster virus were low. Only 9.1% of patients with psoriasis and information about their vaccination status against the three viruses in focus had completed the varicella zoster virus vaccination, and 4.9% had only one of the two recommended doses. We found expected differences in vaccination rates by patient age: Older patients were significantly more likely to have been completely vaccinated compared to patients aged < 60 (*p* ≤ 0.001) and were higher in rates compared to RKI data (almost 3-fold higher in study population: 21.9% vs. 7.7%). Other subgroups of disease severity or treatment did not reveal significant differences. Again, patients with JAK inhibitors as systemic medication had higher rates of completed zoster vaccination (Table 3).

### 3.4. Vaccination in Patients with Atopic Dermatitis

The subgroup of patients with atopic dermatitis and information about their vaccination status comprised 1020 patients. A total of 90.2% of patients had completed the vaccination against SARS-CoV-2, with two basic vaccinations and at least one additional booster; 7.3% of them had at least one dose; and only 2.5% of the patients had no vaccination against SARS-CoV-2. The rate of complete vaccination was significantly higher in older patients. Patients with systemic biologic medication had higher vaccination rates against SARS-CoV-2 than patients with JAK inhibitors or other systemic therapies.

In the same subgroup of patients with atopic dermatitis and information about their vaccination status, 48.8% of the patients had at least one preseasonal vaccination against influenza virus, with older patients aged 60 and above having a higher rate (*p* = 0.001). Patients with a higher grade of skin inflammation had a significantly lower rate than patients with milder disease (*p* = 0.008). Within rates of complete vaccination against influenza virus, we found no differences in patients without and with systemic treatment.

Patients with atopic dermatitis had low vaccination rates against varicella zoster virus. A total of 9.2% of this subgroup had completed the varicella zoster virus vaccination, and 4.9% had only one of the two recommended doses. Again, we found significantly higher rates of complete vaccination in patients aged 60 years and older compared to younger patients (*p* = 0.001). Systemic treatment and disease severity did not seem to affect vaccination rates (Table 4).

The subgroup of patients with both psoriasis and atopic dermatitis included only 78 patients (Figure 1) and was therefore not analyzed in subgroups.

### 3.5. Vaccination in Patients with Other Skin Diseases

The last subgroup of diagnoses included patients with chronic inflammatory skin diseases other than psoriasis and/or atopic dermatitis (n = 2369), and we had no clinical scores like PASI or EASI for the severity of the diseases in this analyzed heterogeneous group. This group consisted of patients with various chronic inflammatory diseases, e.g., hidradenitis suppurativa, lupus erythematodes, pyoderma gangrenosum, vitiligo, and various others, all with the same request for a consultation in our specialized outpatient clinics for systemic treatments due to the severity of the disease. Therefore, we included this group in our analysis. In this group of skin diseases other than psoriasis and/or atopic dermatitis, vaccination rates against SARS-CoV-2 were 89.5% for complete vaccination. Patients aged 60 and above showed significantly higher rates of complete vaccination against SARS-CoV-2 (95.9% vs. 87.1%, *p* = 0.001), influenza virus (74.3% vs. 41.7%, *p* = 0.001), and varicella zoster virus (22.4% vs. 4.7%, *p* = 0.001) compared to patients aged < 60 years. In patients without systemic treatment, we found significantly higher rates of complete vaccination against influenza virus (*p* = 0.020) and varicella zoster virus (*p* = 0.018), but no differences against SARS-CoV-2 (*p* = 0.163). The rates of complete vaccination in patients receiving biologics were 90.5% against SARS-CoV-2, 47.6% against influenza virus, and 6.9% against varicella zoster virus; in patients receiving JAK inhibitors the rates were 76.2%, 50.0%, and 36.8%, respectively; and in patients with other systemic treatment the rates were 85.7%, 61.6%, and 22.2%, respectively (Table 5).

## 4. Discussion

Following guidelines for the treatment of immunosuppressed patients, routine care for these patients should include screenings for infections as well as preventive vaccinations [3,4,10,14,15].

Preparation of patients for immunomodulatory systemic therapies should include information on preventive vaccination to reduce the risk of adverse events such as respiratory tract infections with biologic treatments or reactivation of varicella zoster virus with JAK inhibitors. The vaccination should preferably be carried out before starting systemic treatment. In our patients who came for counselling before or during systemic treatment, vaccination rates against SARS-CoV-2, varicella zoster viruses, or influenza viruses were not satisfying.

In Germany, vaccinations are routinely performed by the general practitioners of the respective patients, and a referral to us for a discussion about systemic therapy of a chronic inflammatory disease should also set a focus on the prevention of communicable diseases. Our data show higher vaccination rates for our patients age 60 and above compared to the German population of the same age group, who have an explicit recommendation for vaccination against influenza virus, varicella zoster virus, and SARS-CoV-2 virus [16]. Every year, RKI Germany reports recent vaccination rates and appeals to the population to use this preventive means. This is unfortunately not reflected by succeeding with increasing vaccination rates for the majority of the focus group.

The awareness of physicians and patients of the importance of preventive vaccinations for patients with immunomodulatory treatments or autoimmune diseases increased with the pandemic, and our team integrated more information about preventive measures, including vaccinations, in our health care for outpatients. Although this was not consistently performed or documented for all patients, we retrieved data on the majority of cases for this analysis out of our regular outpatient clinics. A total of 675 of 8040 files analyzed for this paper did not contain documented information about vaccinations against SARS-CoV-2, influenza virus, or varicella zoster virus which means that this information was not received or documented by the physician during the visit and is a field for improvement for our team, too. Information about vaccination against varicella zoster virus or influenza virus in particular was unknown in a higher number of cases than against SARS-CoV-2 and may reflect the awareness in the average population of COVID-19 disease at that time point. Another problem deriving from this retrospective analysis of routine data is the lack of reconfirmation for orally communicated vaccinations by documented vaccination certificates, which might have led to incorrect information from patients. During the pandemic, vaccination against SARS-CoV-2 was strongly emphasized in Germany, and patients without SARS-CoV-2 vaccination were tasked with explaining this lack of protection. Avoiding this might have led to incorrect statements about their vaccination status. What is more, patients do not necessarily recall vaccinations properly and can mix up names.

At the time of consultation, the majority of patients in all four subgroups were already under systemic therapies, with low scores for disease activity (PASI, EASI) and better quality of life (reflected in the DLQI), but possibly higher risk of infections at the same time.

The analyzed data showed a low vaccination rate for SARS-CoV-2, influenza virus, and varicella zoster virus in our patient cohort overall, with a 79.7% vaccination rate against SARS-CoV-2, 49.7% against influenza virus, and 9.2% against varicella zoster virus. The rates were higher for the vulnerable patient group age 60 and above, with 73.8% against influenza virus, 89.9% against SARS-CoV-2, and 22.3% against varicella zoster virus, compared to the German population of the same age group. These results have to be interpreted with care, always reflecting the limitation of our retrospective analyses of documented oral communications between patients and physicians. The rates are still too low to reach satisfying ranges, and a high proportion of our patients with chronic inflammatory skin diseases age 60 and above are not covered by vaccination against these three viruses.

Only for patients with psoriasis did we see significantly higher vaccination rates against SARS-CoV-2 in patients with systemic medication. All other groups appeared not to be influenced by treatment status, and a field of improvement was demonstrated for these vulnerable patient groups. This correlates with findings of patients with other diseases and similar immunomodulatory treatments [17,18].

Patients under therapeutic treatment with biologics should receive information about susceptibility to respiratory infections, especially in terms of TNF-alpha inhibitors, because the rates are slightly increased [19,20]. An acute infection normally results in an interruption of systemic treatment, with the risk of increased inflammatory activity of the disease, and should be avoided if possible. In our analysis, we did not recognize an influence of the choice of systemic medication on the rates of preventive vaccinations against SARS-CoV-2 or influenza virus.

Especially for immunomodulating systemic therapies, there exist explicit recommendations for vaccinations against varicella zoster virus, reflecting study data and published experiences, with higher rates of reactivation of varicella zoster virus with treatment [6,21,22]. Since the first approval of a zoster vaccine with a live vaccine in 2017 and the availability of a non-live vaccine since 2021, a vaccination was also possible for patients already under immunomodulation, but the rates are still low. The STIKO in Germany recommends vaccination with the non-live vaccine for persons from age 60 and in immunocompromised patients from age 50 [6]. This influences the refundability by government insurance and therefore has a significant impact on physicians’ treatment decisions, especially in the vaccination of younger patients with chronic inflammatory skin diseases receiving systemic treatments.

The vaccination rates against varicella zoster virus for older patients age 60 and above in our study were markedly higher than in the German population of same age (22.3% vs. 7.7%). However, they are still too low for effective population protection [6,21].

In 2021, with the availability of SARS-CoV-2 vaccinations, there was special attention from the media and the world population on this topic, and this is also reflected by higher rates of vaccinations in our patient cohort than against influenza virus. Patients overall and patients age 60 and above had higher rates than in the average population, but a significant number of patients was still not covered by this preventive measure.

## 5. Conclusions

Vaccination rates among our patients who came for counselling before or during systemic treatment were low. Preventive vaccination rates against SARS-CoV-2 were higher than against influenza virus and remarkably low for varicella zoster virus, and, compared to the average German population of the same age, not significantly higher.

The preparation of patients for immunomodulatory systemic therapies should include information on preventive vaccinations to reduce the risk of adverse events such as respiratory tract infections with biologic treatments or reactivation of the varicella zoster virus with JAK inhibitors. Vaccination should preferably be given before starting systemic treatment, but most of the vaccinations are non-live vaccines and therefore approved for use also in immunocompromised conditions, with recommendations of application intervals for optimized efficacy.

In general practitioner care in particular, having at least a contact for referral to us with questions about systemic treatment of an autoimmune chronic inflammatory skin disease offers the opportunity to provide information about recommended vaccinations.

We suggest informing patients about preventive vaccinations before and during systemic immunomodulatory treatments and emphasize the need for active communication in this vulnerable patient group.

### Limitations

Our data were retrieved retrospectively from the files of our specialized outpatient clinics. We excluded files without any documentation about communication about vaccinations, which does not necessarily mean that the patient lacked vaccination.

We set the focus on three vaccinations, two for the most commonly practiced vaccinations against respiratory viruses and one against varicella zoster virus, as a focus for new medications like JAK inhibitors, which does not mean that vaccinations against further infections like RSV or pneumococci have less importance.

Anamnestic data based on oral communication and no vaccination logs were included in the visit files, which might have influenced the actual vaccination rates due to vaccinations that the patient did not remember or could not name. On the other hand, patients might have stated having the vaccination against SARS-CoV-2 without actual having the vaccination to avoid disadvantages like a postponed visit.

We did not include in this analysis information about the frailty of the patients, further medications, comorbidities, or other immunocompromising conditions that might have influenced decisions about vaccinations.

This is a cross-sectional study. We did not include longitudinal comparisons of vaccination rates before and after the start of the pandemic.

Any comparison with the PSO + AD group should be interpreted with caution. The estimated marginal means of PSO + AD were very similar to the other groups. However, the group was too small (and the confidence intervals too wide) to make significant differences visible compared to groups at least 13 times larger.

## Figures and Tables

**Table 1:** Patients’ sociodemographic and disease-related characteristics at the time of inclusion in the analysis during their visit to the outpatient clinic for chronic inflammatory skin diseases from December 2021 to December 2022. Comparison was performed using ANOVA and post-hoc Tamhane-T2. Significant differences between subgroups resulting from post-hoc tests are shown in the last column. M, mean; SD, standard deviation; PASI, Psoriasis Area and Severity Index; DLQI, Dermatology Life Quality Index; EASI, Eczema Area and Severity Index, AD, atopic dermatitis, PSO, psoriasis.

		Patients with Psoriasis (n = 3898)	Patients with Atopic Dermatitis (n = 1020)	Patients with Psoriasis and Atopic Dermatitis (n = 78)	Patients with Other Diagnoses (n = 2369)	*p*-Value
Age (years), M ± SD Median (range)		49.0 ± 15.2 49.0 (18.0–91.0)	43.4 ± 16.5 40.0 (18.0–92.0)	48.3 ± 14.4 49.5 (18.0–74.0)	49.6 ± 16.3 50.0 (18.0–89.0)	0.027 AD < (PSO, PSO + AD, others)
Gender n (%)	Male	2351 (60.3%)	514 (50.4%)	45 (57.7%)	1332 (56.2%)	0.010 % Male: PSO > (AD, others)
	Female	1545 (39.6%)	506 (49.6%)	33 (42.3%)	1035 (43.7%)	
	Non-binary	1 (0.0%)	-	-	1 (0.0%)	
	Missing	1 (0.0%)	-	-	1 (0.0%)	
Patients starting or being on systemic therapy		3232 (82.9%)	695 (68.1%)	54 (69.2%)	1565 (66.1%)	0.001 PSO > (AD, others)
Patients without systemic therapy		666 (17.1%)	325 (31.9%)	24 (30.8%)	804 (33.9%)	0.001 PSO < (AD, others)
PASI ≥ 10		106 (2.7%)	NA	3 (3.8%)	NA	NA
PASI < 10		3792 (97.3%)	NA	75 (96.2%)	NA	
DLQI ≥ 10 *		490 (12.6%)	233 (22.8%)	15 (19.2%)	337 (14.2%)	0.001
DLQI < 10		3330 (85.4%)	768 (75.3%)	61 (78.2%)	1892 (79.9%)	AD > (PSO, others)
EASI ≥ 7		NA	80 (7.8%)	5 (6.4%)	NA	NA
EASI < 7		NA	940 (92.2%)	73 (93.6%)	NA	

* Subgroups do not sum up to 100% due to missing data.

**Table 2:** Comparison of vaccination rates for influenza virus, SARS-CoV-2, and varicella zoster virus in a population of German adults aged 60 years and older from 2021 to 2022 and the present study population from December 2021 to December 2022, specifically considering people older than 60 years.

Vaccination Rates Valid %(n; Missing)	In Study Population	In Study Population (Adults 60 Years and Older)	In Germany 2021/2022 (Adults 60 Years and Older) *
Influenza virus (One preseasonal application)	49.7% (2862; 1602)	73.8% (1041; 427)	43.3%
SARS-CoV-2 (two applications and at least one booster)	79.7% (5869; 0)	89.9% (1652; 0)	61.8%
Varicella zoster virus (two applications)	9.2% (436; 2641)	22.3% (254; 700)	7.7%

* [12] Archive/2022-0101_Deutschland_Impfquoten_COVID-19.csv. [13] Vaccination in patients with psoriasis.

**Table 3:** Patients with psoriasis and or psoriatic arthritis and documented vaccinations against SARS-CoV-2 (no = 0 vaccinations, incomplete = 1–2 vaccinations, complete = 3 or more vaccinations or immunization by infection, unknown = no answer), influenza virus (no = 0 vaccinations, yes = 1 vaccination, unknown = no answer), or varicella zoster virus (no = 0 vaccinations, incomplete = 1 vaccination, complete = 2 vaccinations). Significant differences are marked in bold (Fisher’s exact test). PASI, Psoriasis Area and Severity Index; JAK, Janus kinase.

Patients with Information on Vaccination Status Regarding SARS-CoV-2, Influenza Virus, and/or Varicella Zoster Virus; n = 7365		Vaccination against SARS-CoV-2 Valid % (n)				Vaccination against Influenza Virus Valid % (n)			Vaccination against Varicella Zoster Virus Valid % (n)			
		No	Incomplete	Complete	Missing Data	No	Yes	Missing Data	No	Incomplete	Complete	Missing Data
Patients with psoriasis;		3.1% (119)	9.5% (365)	87.4% (3352)	62	50.7% (1522)	49.3% (1522)	897	86.0% (2127)	4.9% (121)	9.1% (226)	1424
n = 3898												
Age < 60; n = 2912		3.7% (107)	11.4% (328)	**84.9% (2440)**	37	59.2% (1321)	**40.8% (1321)**	679	92.3% (1718)	2.7% (51)	**4.9% (92)**	1051
Age ≥ 60; n = 986		1.2% (12)	3.9% (37)	**94.9% (912)**	25	26.2% (201)	**73.8% (201)**	218	66.7% (409)	11.4% (70)	**21.9% (134)**	373
PASI ≥ 10; n = 106		4.8% (5)	17.3% (18)	**77.9% (81)**	2	61.4% (51)	38.6% (51)	23	83.3% (55)	7.6% (5)	9.1% (6)	40
PASI < 10; n = 3792		3.1% (114)	9.3% (347)	**87.6% (3271)**	60	50.4% (1471)	49.6% (1471)	874	86.0% (2072)	4.8% (116)	9.1% (220)	1384
No systemic therapy;		3.0% (20)	13.1% (86)	**83.9% (552)**	8	50.6% (263)	49.4% (263)	146	86.0% (374)	5.1% (22)	9.0% (39)	231
n = 666												
Systemic therapy;		3.1% (99)	8.8% (279)	**88.1% (2800)**	54	50.7% (1259)	49.3% (1259)	751	86.0% (1753)	4.9% (99)	9.2% (187)	1193
n = 3232												
Systemic therapy *;	Biologic therapy; **	3.2% (95)	9.0% (267)	87.8% (2604)	52	51.8% (1194)	48.2% (1194)	713	86.8% (1651)	4.8% (91)	8.4% (160)	1116
n = 3232	n = 3018											
	JAK inhibitor;	-	-	100.0% (18)	-	31.3% (5)	68.8% (5)	2	58.3% (7)	-	41.7% (5)	6
	n = 18											
	Other systemic therapy; n = 267	1.5% (4)	7.7% (20)	90.8% (237)	6	37.8% (79)	62.2% (79)	58	73.3% (121)	7.3% (12)	19.4% (32)	102

* Numbers are not additive since patients can receive more than one systemic treatment at the same time. ** Biologic therapeutics included TNF-alpha inhibitors, IL17 inhibitors, and IL23 or Il12/23 inhibitors.

**Table 4:** Patients with atopic dermatitis and documented vaccinations against SARS-CoV-2 (no = 0 vaccinations, incomplete = 1–2 vaccinations, complete = 3 or more vaccinations or immunization by infection, unknown = no answer), influenza virus (no = 0 vaccinations, yes = 1 vaccination, unknown = no answer), or varicella zoster virus (no = 0 vaccinations, incomplete = 1 vaccination, complete = 2 vaccinations). Significant differences are marked in bold (Fisher’s exact test). EASI, Eczema Area and Severity Index; JAK, Janus kinase.

Patients with Information on Vaccination Status Regarding SARS-CoV-2, Influenza Virus, and/or Varicella Zoster Virus; n = 7365		Vaccination against SARS-CoV-2Valid % (n)				Vaccination against Influenza VirusValid % (n)			Vaccination against Varicella Zoster VirusValid % (n)			
		No	Incomplete	Complete	Missing Data	No	Yes	Missing Data	No	Incomplete	Complete	Missing Data
Patients with atopic dermatitis		2.5% (25)	7.3% (74)	90.2% (910)	11	51.2% (421)	48.8% (421)	197	85.9% (581)	4.9% (33)	9.2% (62)	344
n = 1020												
Age < 60; n = 860		2.6% (22)	8.4% (72)	**89.0% (759)**	7	55.2% (392)	**44.8% (392)**	150	90.7% (518)	3.3% (19)	**6.0% (34)**	289
Age ≥ 60; n = 160		1.9% (3)	1.3% (2)	**96.8% (151)**	4	25.7% (29)	**74.3% (29)**	47	60.0% (63)	13.3% (14)	**26.7% (28)**	55
EASI ≥ 7; n = 80 (100%)		6.4% (5)	7.7% (6)	85.9% (67)	2	66.7% (46)	**33.3% (46)**	11	87.9% (51)	5.2% (3)	6.9% (4)	22
EASI < 7; n = 940 (100%)		2.1% (20)	7.3% (68)	90.5% (843)	9	49.7% (375)	**50.3% (375)**	186	85.8% (530)	4.9% (30)	9.4% (58)	322
No systemic therapy;		3.8% (12)	6.6% (21)	89.7% (287)	5	53.1% (138)	46.9% (138)	65	84.6% (192)	4.4% (10)	11.0% (25)	98
n = 325												
Systemic therapy;		1.9% (13)	7.7% (53)	90.4% (623)	6	50.3% (283)	49.7% (283)	132	86.6% (389)	5.1% (23)	8.2% (37)	246
n = 695												
Systemic therapy *;	Biologic therapy; **	1.5% (9)	7.7% (45)	90.8% (533)	3	53.1% (254)	46.9% (254)	112	90.3% (344)	3.9% (15)	5.8% (22)	209
n = 695	n = 590											
	JAK inhibitor;	4.0% (4)	8.1% (8)	87.9% (87)	3	34.1% (28)	65.9% (28)	20	67.2% (45)	10.4% (7)	22.4% (15)	35
	n = 102											
	Other systemic therapy; n = 7	-	14.3% (1)	85.7% (6)	-	14.3% (1)	85.7% (1)	-	80.0% (4)	-	20.0% (1)	2

* Numbers are not additive since patients can receive more than one systemic treatment at the same time. ** Biologic therapeutics included TNF-alpha inhibitors, IL17 inhibitors, and IL23 or Il12/23 inhibitors.

**Table 5:** Patients with diagnoses other than psoriasis (arthritis) and atopic dermatitis and documented vaccinations against SARS-CoV-2 (no = 0 vaccinations, incomplete = 1–2 vaccinations, complete = 3 or more vaccinations or immunisation by infection, unknown = no answer), influenza virus (no = 0 vaccinations, yes = 1 vaccination, unknown = no answer), or varicella zoster virus (no = 0 vaccinations, incomplete = 1 vaccination, complete = 2 vaccinations). Significant differences are marked in bold (Fisher’s exact test). JAK, Janus kinase.

Patients with Information on Vaccination Status Regarding SARS-CoV-2, Influenza Virus, and/or Varicella Zoster Virus; n = 7365		Vaccination against SARS-CoV-2 Valid % (n)				Vaccination against Influenza Virus Valid % (n)			Vaccination against Varicella Zoster Virus Valid % (n)			
		No	Incomplete	Complete	Missing Data	No	Yes	Missing Data	No	Incomplete	Complete	Missing Data
Patients with other diagnosis		2.9% (68)	7.5% (175)	89.5% (2082)	44	49.4% (927)	50.6% (927)	492	86.2% (1317)	4.3% (66)	9.4% (144)	842
n = 2369												
Age < 60; n = 1697		3.6% (61)	9.3% (155)	**87.1% (1456)**	25	58.3% (795)	**41.7% (795)**	334	93.1% (1040)	2.2% (25)	**4.7% (52)**	580
Age ≥ 60; n = 672		1.1% (7)	3.1% (20)	**95.9% (626)**	19	25.7% (132)	**74.3% (132)**	158	67.6% (277)	10.0% (41)	**22.4% (92)**	262
No systemic therapy;		3.2% (25)	8.4% (66)	88.4% (696)	17	45.7% (301)	**54.3% (301)**	145	83.6% (437)	4.6% (24)	**11.9% (62)**	281
n = 804												
Systemic therapy;		2.8% (43)	7.1% (109)	90.1% (1386)	27	51.4% (626)	**48.6% (626)**	347	87.6% (880)	4.2% (42)	**8.2% (82)**	561
n = 1565												
Systemic therapy *;	Biologic therapy; **	2.9% (41)	6.5% (92)	90.5% (1273)	25	52.4% (581)	47.6% (581)	322	89.0% (814)	4.2% (38)	6.9% (63)	516
n = 1565	n = 1431											
	JAK inhibitor;	0.0% (0)	23.8% (5)	76.2% (16)	0	50.0% (10)	50.0% (10)	1	52.6% (10)	10.5% (2)	36.8% (7)	2
	n = 21											
	Other systemic therapy; n = 147	1.4% (2)	11.1% (16)	87.5% (126)	3	38.2% (42)	61.8% (42)	37	74.4% (67)	3.3% (3)	22.2% (20)	57

* Numbers are not additive since patients can receive more than one systemic treatment at the same time. ** Biologic therapeutics included TNF-alpha inhibitors, IL17 inhibitors, and IL23 or Il12/23 inhibitors.

## Data Availability

The data that support the findings of this study are not openly available due to reasons of sensitivity and are available from the corresponding author upon reasonable request.

## References

[1] Atzeni F., Batticciotto A., Masala I.F., Talotta R., Benucci M., Sarzi-Puttini P. (2016). Infections and Biological Therapy in Patients with Rheumatic Diseases. Isr. Med. Assoc. J..

[2] Gabay C., Bel M., Combescure C., Ribi C., Meier S., Posfay-Barbe K., Grillet S., Seebach J.D., Kaiser L., Wunderli W. (2011). Impact of synthetic and biologic disease-modifying antirheumatic drugs on antibody responses to the AS03-adjuvanted pandemic influenza vaccine: A prospective, open-label, parallel-cohort, single-center study. Arthritis Rheum..

[3] Wagner N., Assmus F., Arendt G., Baum E., Baumann U., Bogdan C., Burchard G., Föll D., Garbe E., Hecht J. (2019). Impfen bei Immundefizienz: Anwendungshinweise zu den von der Ständigen Impfkommission empfohlenen Impfungen. (IV) Impfen bei Autoimmunkrankheiten, bei anderen chronisch-entzündlichen Erkrankungen und unter immunmodulatorischer Therapie. Bundesgesundheitsblatt Gesundheitsforschung Gesundheitsschutz.

[4] Elkayam O. (2018). SP0158 Update of eular recommendations for vaccination of patients with autoimmune inflammatory rheumatic diseases. Proceedings of the Annual European Congress of Rheumatology, EULAR 2018.

[5] Gelfand J.M., Armstrong A.W., Bell S., Anesi G.L., Blauvelt A., Calabrese C., Dommasch E.D., Feldman S.R., Gladman D., Kircik L. (2020). National Psoriasis Foundation COVID-19 Task Force Guidance for Management of Psoriatic Disease During the Pandemic: Version 1. J. Am. Acad. Dermatol..

[6] Robert Koch-Institut (2018). Wissenschaftliche Begründung zur Empfehlung einer Impfung mit dem Herpes Zoster-Subunit-Totimpfstoff.

[7] Gisondi P., Facheris P., Dapavo P., Piaserico S., Conti A., Naldi L., Cazzaniga S., Malagoli P., Costanzo A. (2020). The impact of the COVID-19 pandemic on patients with chronic plaque psoriasis being treated with biological therapy: The Northern Italy experience. Br. J. Dermatol..

[8] Gresham L.M., Marzario B., Dutz J., Kirchhof M.G. (2021). An evidence-based guide to SARS-CoV-2 vaccination of patients on immunotherapies in dermatology. J. Am. Acad. Dermatol..

[9] Lebwohl M., Rivera-Oyola R., Murrell D.F. (2020). Should biologics for psoriasis be interrupted in the era of COVID-19?. J. Am. Acad. Dermatol..

[10] Augustin M., Reich K., Mrowietz U. Verfahrensweise bei der Systemtherapie von Patienten mit Psoriasis Während der Pandemischen Phase von SARS-CoV-2 (Coronavirus): Letter of Recommendations. https://www.bvdd.de/aktuelles-presse/newsroom/alle-nachrichten/details/empfehlungen-zu-systemtherapien-bei-schuppenflechte/.

[11] Radtke M.A., Schäfer I., Glaeske G., Jacobi A., Augustin M. (2017). Prevalence and comorbidities in adults with psoriasis compared to atopic eczema. J. Eur. Acad. Dermatol. Venereol..

[12] Rieck T., Steffen A., Feig M., Siedler A. (2021). Impfquoten bei Erwachsenen in Deutschland—Aktuelles aus der KV-Impfsurveillance. Epid. Bull..

[13] Robert Koch-Institut (2024): COVID-19-Impfungen in Deutschland, Berlin: Zenodo. https://zenodo.org/records/12697471.

[14] Lopez A., Mariette X., Bachelez H., Belot A., Bonnotte B., Hachulla E., Lahfa M., Lortholary O., Loulergue P., Paul S. (2017). Vaccination recommendations for the adult immunosuppressed patient: A systematic review and comprehensive field synopsis. J. Autoimmun..

[15] Winthrop K.L., Mariette X., Silva J.T., Benamu E., Calabrese L.H., Dumusc A., Smolen J.S., Aguado J.M., Fernández-Ruiz M. (2018). ESCMID Study Group for Infections in Compromised Hosts (ESGICH) Consensus Document on the safety of targeted and biological therapies: An infectious diseases perspective (Soluble immune effector molecules II: Agents targeting interleukins, immunoglobulins and complement factors). Clin. Microbiol. Infect..

[16] Vygen-Bonnet S., Schlaberg J., Koch J. (2022). Rolle, Arbeitsweise und Empfehlungen der Ständigen Impfkommission (STIKO) im Kontext der COVID-19-Pandemie. Bundesgesundheitsblatt Gesundheitsforschung Gesundheitsschutz.

[17] Hmamouchi I., Winthrop K., Launay O., Dougados M. (2015). Low rate of influenza and pneumococcal vaccine coverage in rheumatoid arthritis: Data from the international COMORA cohort. Vaccine.

[18] Loubet P., Kernéis S., Groh M., Loulergue P., Blanche P., Verger P., Launay O. (2015). Attitude, knowledge and factors associated with influenza and pneumococcal vaccine uptake in a large cohort of patients with secondary immune deficiency. Vaccine.

[19] Lahiri M., Dixon W.G. (2015). Risk of infection with biologic antirheumatic therapies in patients with rheumatoid arthritis. Best Pract. Res. Clin. Rheumatol..

[20] Ling J., Koren G. (2016). Challenges in vaccinating infants born to mothers taking immunoglobulin biologicals during pregnancy. Expert Rev. Vaccines.

[21] Marra F., Lo E., Kalashnikov V., Richardson K. (2016). Risk of Herpes Zoster in Individuals on Biologics, Disease-Modifying Antirheumatic Drugs, and/or Corticosteroids for Autoimmune Diseases: A Systematic Review and Meta-Analysis. Open Forum Infect. Dis..

[22] Tran C.T., Ducancelle A., Masson C., Lunel-Fabiani F. (2017). Herpes zoster: Risk and prevention during immunomodulating therapy. Jt. Bone Spine.

